# Nutritional Features and Bread-Making Performance of Wholewheat: Does the Milling System Matter?

**DOI:** 10.3390/foods9081035

**Published:** 2020-08-01

**Authors:** Maria Ambrogina Pagani, Debora Giordano, Gaetano Cardone, Antonella Pasqualone, Maria Cristina Casiraghi, Daniela Erba, Massimo Blandino, Alessandra Marti

**Affiliations:** 1 Department of Food, Environmental and Nutritional Sciences (DeFENS), Università degli Studi di Milano, via G. Celoria 2, 20133 Milan, Italy; ambrogina.pagani@unimi.it (M.A.P.); gaetano.cardone@unimi.it (G.C.); maria.casiraghi@unimi.it (M.C.C.); daniela.erba@unimi.it (D.E.); 2 Department of Agricultural, Forest and Food Sciences (DISAFA), Università degli Studi di Torino, Largo P. Braccini 2, 10095 Grugliasco (TO), Italy; debora.giordano@unito.it; 3 Food Science and Technology Unit, Department of Science of Soil, Università degli Studi di Bari, via Amendola, 165/A, 70126 Bari, Italy; antonella.pasqualone@uniba.it

**Keywords:** wholewheat flour, stone milling, roller milling, bioactive compounds, bread, pigmented wheat, dough rheology

## Abstract

Despite the interest in stone-milling, there is no information on the potential advantages of using the resultant wholegrain flour (WF) in bread-making. Consequently, nutritional and technological properties of WFs obtained by both stone- (SWF) and roller-milling (RWF) were assessed on four wheat samples, differing in grain hardness and pigment richness. Regardless of the type of wheat, stone-milling led to WFs with a high number of particles ranging in size from 315 to 710 μm), whereas RWFs showed a bimodal distribution with large (>1000 μm) and fine (<250 μm) particles. On average, the milling system did not affect the proximate composition and the bioactive features of WFs. The gluten aggregation kinetics resulted in similar trends for all SWFs, with indices higher than for RWFs. The effect of milling on dough properties (i.e., mixing and leavening) was sample dependent. Overall, SWFs produced more gas, resulting in bread with higher specific volume. Bread crumb from SWF had higher lutein content in the wheat *cv* rich in xanthophylls, while bread from RWF of the blue-grained *cv* had a moderate but significantly higher content in esterified phenolic acids and total anthocyanins. In conclusion, there was no relevant advantage in using stone- as opposed to roller-milling (and vice versa).

## 1. Introduction

Whole grains have been (and still are in several countries) the most important energy source of mankind. They constitute a valuable and inexpensive source of numerous nutrients and phytochemicals, including fiber, phenolic compounds, minerals, and vitamins, mainly located in the germ and bran regions [1,2]. Although a universally accepted definition of whole grain has yet to be formulated [3], it is widely recognized that a whole grain product must contain bran, germ and endosperm in the same proportions as in the original, native grain [3]. Whatever the official definition is, the relationship between the consumption of whole grain foods and a lower incidence of diseases in the Occident has been suggested by numerous epidemiological studies [4,5,6,7,8].

Thanks to public institutions (governments and health organizations) and extensive promotional campaigns, consumers nowadays are fully aware of the numerous health benefits associated with the consumption of products made from whole grain flour (WF). Consequently, the demand for wholegrain products—in particular for staple foods such as bread and pasta—has been constantly growing [9]. This trend is recognizable even in countries where products made from refined flour have always been preferred for their undisputed superior sensory characteristics.

At present, two milling processes are mainly used to produce WF from wheat: (i) single–stream milling, and (ii) multiple-stream milling with recombination [10]. The former—where stone mills are used—is the world’s oldest system for flour production. The stone mill is quite a simple machine, formed by two horizontal and overlapping grinding stones where the upper revolves while the other (bedstone) is stationary [11]. Grains are fed in the gap between the two stones and undergo shear, compression and abrasion forces. The ground fractions stay together during the entire milling process, and they are collected (with an extraction rate of 100%) at the bottom of the bedstone without separation according to particle size or composition [12].

Multiple-stream milling became established in the mid-1800s [11]. According to this process, wheat grains are progressively ground by means of a sequence of cast iron roller mills followed by sieving and sifting of the ground material [13]. Due to differences in composition, bran, germ and endosperm exhibit different friability and breakage patterns, thus facilitating the separation of bran and germ from flour, which derives from the endosperm. The separation of the three regions is enhanced by conditioning the kernels before milling [11,13]. The flour extraction rate is usually about 70% [12]. Even this milling process may lead to a WF if the different “fraction streams”—originating with the repeated grinding and sieving steps—are gathered at the end of the process. The extraction rate of the recombined WF should be about 100%, and the endosperm, germ and bran should be present in the same ratio as in the native whole grain [10]. Roller milling guarantees higher productivity, flexibility and more constant results over time; consequently, this process is highly preferred for industrial applications [13,14]. Moreover, the separation of bran and germ fractions during the process allows further treatments before recombination, such as mild heating and/or bran grinding, improving both technological performance and storage of the WF [13,15].

On the other hand, stone milling has been rediscovered in recent years by small farmers and bread-making artisans as it requires relatively low capital inputs [16]. Certainly, the simplicity and cheapness of the process (only one operation; grain tempering is not mandatory) also makes it suitable for household milling, favoring whole grain consumption for some segments of population [14]. Nevertheless, despite these advantages, stone milling not only is characterized by a lower yield but may also worsen the rheological properties of WF due to varying degrees of heat development [16,17] according to the type of small-scale mills used [14]. The negative effects of stone milling can be reduced by choosing suitable wheat *cultivars (cvs)* and/or farming procedures, as suggested by Gélinas et al. [18].

Despite the great interest in stone milling and increasing demands for WF-produced food, few studies have compared the effects of stone and roller milling on the same wheat varieties to identify and evaluate analogies and differences between the two kinds of WF [15,19]. Some research has found similarities in proximate chemical composition and phenolic profile of WF from stone milling (SWF) and roller milling (RWF) [17,20,21]. On the contrary, by promoting high heat development due to friction, compression and shear phenomena, stone milling leads to a significant loss in aminoacids and unsaturated fatty acids [17]. This worsening can affect not only the nutritional aspects of flour but also its technological properties, due to a partial denaturation of gluten proteins, relevant starch damage and/or differences in particle size distribution [15,17,20]. Specifically, SWFs exhibited lower pasting properties, higher water absorption, and lower stability compared to RWFs [14,21].

Regarding the relationship between milling process and bread quality, the data are not in agreement. Liu et al. [21] emphasized that RWFs exhibited the best steam-bread making performance, while according to Kihlberg et al. [22], bread produced using RWF was characterized by regular shape but higher compactness. Nevertheless, differences in bread volume in WFs obtained by the two types of milling can be resolved by adopting sourdough fermentation [14].

The few studies published so far on the effects of the milling process on WF and bread properties obtained by the same type of wheat presented no univocal findings and prompted our study to aim at giving a complete overview of behavior–from milling to baking–of WFs from four types of common wheat (*T. aestivum* L.), all belonging to the bread-making class and characterized by high protein content. Beyond these common traits, the four wheat samples differed in protein strength and physical structure of kernels–hard, semi-hard and soft endosperm–a property that highly impacts milling behavior and performance [23]. Moreover, two pigmented varieties were distinguished by their richness in bioactive compounds, differently located in the kernel (polyphenols and anthocyanins in the external layers and xanthophylls in the endosperm), whose retention has to be carefully ensured along the whole transformation chain.

## 2. Materials and Methods

### 2.1. Wheat Samples

The present study analyzed the SWF and RWF derived from four common wheat (*Triticum aestivum* L.) samples.

Table 1 compares the main information regarding the wheat samples. The two pigmented wheat *cvs* were chosen for their interesting bioactive compound content [24].

### 2.2. Milling Procedures

An aliquot (70 kg) of each sample was stone-milled (Molino Tomatis; Niella Tanaro, Italy), without kernel conditioning through a single-stream process, without any sifting, to produce SWF. The stone mill used was made of two discs of natural French burrstones, from the district of La Ferté-sous-Jouarre (France). The stones, 1.3 m in diameter, are arranged on a vertical axis, with the upper stone rotating at 70 rpm. The distance between the two stones at their centers was about 1 mm. The opposing surfaces of the stones were subdivided into 10 harps and grooved from the center to the circumference. After the proper cleaning operation, 40 kg of each sample was milled and the flour discarded, in order to reach stable operative conditions (speed, temperature) of the stone mill before sampling. The remaining 30 kg aliquot was then milled, carefully mixed to favor fraction blending and subsampled for rheological and chemical analysis. Another aliquot (10 kg) of grains was conditioned (16 h at 20 °C) till reaching 16% moisture, and then submitted to multiple-stream milling by using a roller mill (Bona lab-scale mill, Labormill 4RB, Monza, Italy). This lab scale mill, equipped with 4 rollers, 0.07 m in diameter and 0.20 m wide were horizontally arranged can simulate the industrial milling process e by separating the different parts of the wheat kernel: the external coarse bran, the intermediate layers (middlings) and the inner endosperm (refined flour). The kernels are milled in three steps: a first break phase passing through fluted rolls and two reducing phases with smooth rolls. Gap settings of the break and reducing rolls were adjusted to 0.4 mm, 0.2 mm and 0.05 mm, respectively. The feed rate was adjusted to about 8 kg/h. The three milling fractions obtained (refined flour, middlings and coarse bran) were recombined to produce the RWF. WF yield was about 100% for both milling processes. Refined flours obtained from roller milling were produced for bread production and functional characterization (see Section 2.3.2).

Samples were stored in a polypropylene bag at 4 °C and under vacuum until used. Samples were used after two weeks of resting.

### 2.3. Methods

#### 2.3.1. Particle Size Distribution

Particle size distribution of SWFs and RWFs was assessed in single by means of an automatic mechanical sifter (AS 200, Retsch GmbH, Düsseldorf, Germany) equipped with 8 sieves: 1000 μm, 800 μm, 710 μm, 500 μm, 425 μm, 315 μm, 250 μm, 160 μm. The test was carried out on 100 g of WF, setting 1.5 mm of oscillation for 5 min.

#### 2.3.2. Chemical Analysis

The moisture content of WFs was determined by means of thermo-balance (MA 210.R, Radwag Wagi Elektroniczne, Radom, Poland), by drying the sample at 130 °C until its weight did not change by 1 mg in 10 s. Ash (AACC 08-01.01) and damaged starch (AACC 76-31.01) contents were evaluated according to AACC standard methods [25]. The amounts of protein (AOAC 34.01.05 No. 925.31), fat (AOAC 31.04.02 No. 963.15), total (AOAC 31.04.02 No. 985.29), soluble and insoluble dietary fiber contents were measured according to AOAC standard methods (AOAC 31.04.02 No. 991.43) [26]. Total and water soluble arabinoxylans were evaluated as previously reported by Manini et al. [27]. Total starch content was calculated as what remained after moisture, protein, ash, fat and total fiber determinations had been accounted for. α-amylase activity was determined according to the AACC method 22-02.01 [25].

The SWF and RWF from each *cv* were analysed for soluble phenolic acids (SPAs) and cell wall-bound phenolic acids (CWBPAs) and total antioxidant capacity. Flours from Bona Vita and Skorpion *cvs* were further analysed for xanthophylls and total anthocyanin content (TAC), respectively. Extraction of phenolic acids and xanthophylls and their quantification by means of RP-HPLC was performed as reported by Giordano et al. [28]. The antioxidant capacity was determined by means of FRAP (Ferric Reducing Antioxidant Power) and the ABTS [2,2′-Azino-bis(3-ethylbenzthiazoline-6-sulfonic acid)] assays adapted into QUENCHER method as described by Serpen et al. [29]. The TAC was determined on samples (1 g) extracted using 8 mL of ethanol acidified with 1 N HCl (85:15, *v/v*) for 30 min. The absorbance was measured after centrifugation at 20,800× *g* for 2 min at 540 nm as reported by Siebenhandl et al. [30]. TAC was expressed as mg cyanidin-3-*O*-glucoside (Cy-3-glc) equivalents/kg of sample (db).

Moisture, ash, protein, fat, soluble and insoluble dietary fiber, total and water soluble arabinoxylans, and amylase activity were evaluated in duplicate, whereas damaged starch content and bioactive compounds in triplicate.

#### 2.3.3. Rheological Properties

Gluten aggregation properties were measured in triplicate by means of the GlutoPeak (Brabender GmbH and Co KG, Duisburg, Germany) device, by using distilled water as solvent; 9:10 flour:solvent ratio, 2750 rpm as paddle speed rate and 35 °C as temperature of water circulating bath. The main indices considered were: (i) maximum torque (the highest consistency value reached, evaluated in GlutoPeak Units, GPU); (ii) peak maximum time (time required to achieve the maximum torque, evaluated in seconds, s); and (iii) aggregation energy (the area under the curve between 15 s before and 15 s after the maximum torque; evaluated in GlutoPeak Equivalents, GPE).

Mixing properties were evaluated in duplicate by using the Farinograph-E (Brabender GmbH and Co KG, Duisburg, Germany) device equipped with a 50 g mixing bowl, according to the standard method ICC 115/1 [31].

Dough leavening properties were determined in single by means of the Rheofermentometer F4 (Chopin Technologies, Villeneuve La Garenne CEDEX, France) device on 315 g of dough at 30 °C for 4.5 h. Dough samples were prepared by mixing flour, in a spiral mixer (Artisan, KitchenAid^®^, Whirlpool, Benton Harbor, USA) with fresh yeast (2 g/100 g of flour; Carrefour, Annecy, France) and salt (NaCl; 1 g/100 g of flour; Candor^®^, Com-Sal s.r.l., Pesaro, Italy). The amount of tap water added, and the kneading time used were previously determined by means of the farinographic test (Table 2).

#### 2.3.4. Bread Preparation and Characterization

Dough samples were prepared from either WFs or refined flours (obtained by roller milling) in the same conditions as those reported for the rheofermentographic test. After kneading, the samples were left to rest for 15 min, then divided into portions of 250 g, modelled in cylindrical shapes, put into aluminum pans (length: 12.5 cm; width: 6 cm; height: 5 cm) and leavened at 30 °C and 70% relative humidity in a combined proofer oven (Self-Cooking Center^®^, Rational International AG, Heerbrugg, Switzerland), until the dough exceeded the top of the baking pans by about 2.5 cm. Then, the leavened dough samples were baked (Self-Cooking Center^®^, Rational International AG, Heerbrugg, Switzerland) at 200 °C for 30 min (85% relative humidity). One baking test was performed, yielding two loaves for each sample.

Two hours after baking, the loaves were characterized in terms of specific volume, through the ratio between the bread volume—evaluated according to the standard method AACC 10-05.01 [25]–and its weight. Bread height was determined by measuring the maximum height of the slice by means of image analysis (Image ProPlus, v6; Media Cybernetics, Inc., Rockville, MD, USA). Loaf specific volume was determined on two loaves, while bread height was evaluated on three central slices of each bread, for a total of six replicates.

SPAs, CWBPAs, total antioxidant capacity, and xanthophylls (only for flour from Bona Vita *cv*) and TAC (only for flour from Skorpion *cv*) were carried out as described in Section 2.3.2, on bread samples obtained from SWF, RWF and refined flours of pigmented *cvs*, which provide a higher AC and the possibility to investigate the impact of milling method also on the bioactive compounds responsible for flour color. Before analysis, bread samples were ground to a fine powder (particle size <300 μm) with a Cyclotec 1093 sample mill (Foss, Padova, Italy). The same grinding procedure was carried out for bread crust (about 3.5 mm thick), and crumb after freeze-drying (−80 °C for 72 h; Alpha 1–2 LD plus; Deltek s.r.l., Naples, Italy). All samples were stored at −25 °C. Bioactive compounds in bread were evaluated in triplicate.

### 2.4. Statistical Analysis

Statistical analysis (t-test) was carried out in order to identify significant differences between SWF and RWF from the same *cv* by using Statgraphics Plus 5.1 (StatPoint Inc., Warrenton, CT, USA) at the 1% (* *p* < 0.01) significance level. Data obtained from the functional characterization of flours and breads were analyzed separately for each *cv* using analysis of variance (ANOVA), by comparing raw material (flour), bread crust and bread crumb obtained from refined flour, SWF and RWF. A 0.01 threshold was used to reject the null hypothesis. The REGW-F test was performed for multiple comparisons, by using SPSS for Windows statistical package, Version 24.0 (SPSS Inc., Chicago, IL, USA).

## 3. Results

### 3.1. Particle Size Distribution

**Figure 1:**
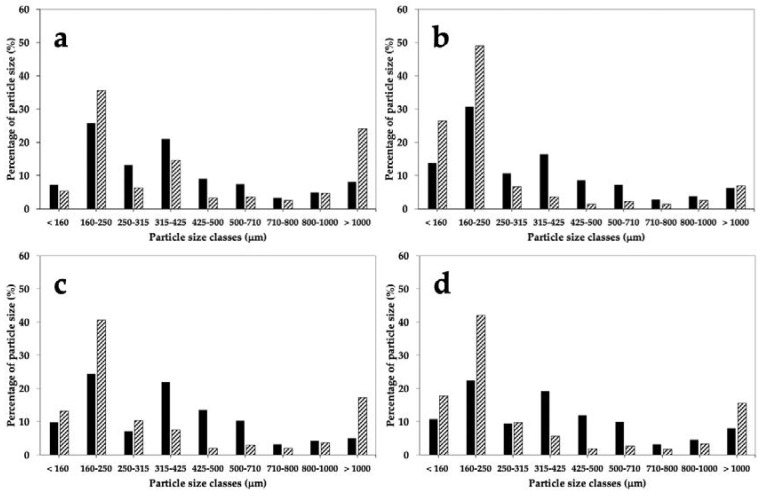
Effect of stone milling (black bars) and roller milling (dash bars) on the particle size distribution of whole grain flours from Bolero *cv* (**a**), CWRS (**b**), Bona Vita *cv* (**c**) and Skorpion *cv* (**d**). CWRS: commercial Canada Western Red Spring Wheat.

Regardless of the type of wheat, stone milling led to WF with a higher number of particles from 315 to 710 μm (from 32.2% for CWRS to 45.9% for Bona Vita *cv*) (Figure 1). On the contrary, in WF obtained by roller milling (i.e., RWFs) such “intermediate” fractions extended from 7% for CWRS to 22% for Bolero *cv*. In addition, recombination of roller milling fractions led to a peculiar, bimodal distribution with large (>1000 μm, mainly composed by bran) and fine (<250 μm) particles. Indeed, the large particles extended from 15% to 25% (except for CWRS) in RWFs, whereas they did not exceed 8% in SWFs. On the other hand, fine particles represented more than 50% (*w/w*) in RWFs, reaching 75% for samples with the highest kernel hardness (i.e., CWRS); whereas this fraction was only 30% of the mass in SWFs.

### 3.2. Chemical Composition and Bioactive Compounds in WFs

The milling system did not significantly affect the chemical composition of WFs, except for the moisture content that decreased by 17% and 11% when Bona Vita and Skorpion *cvs* were milled using the stone milling system (Table 3). Although the fiber content did not change, roller milling caused a slight but significant decrease in the total arabinoxylan content of both CWRS and Bona Vita *cv* (about 19% and 12%, respectively). In contrast, a significant increase in this parameter was found in RWF from Skorpion *cv* (about 70%). As regards the water soluble arabinoxylan fraction, the roller milling system resulted in an increase of about 63% for only Bona Vita *cv* (Table 3). The effect of milling on the bioactive compound concentration was sample and compound dependent. RWFs resulted in a significantly higher content of CWBPAs, CWB-ferulic acid only in Bona Vita and Skorpion *cvs*. A similar trend was observed for CWB-sinapic acid, with a significant difference observed in the CWRS. RWF also showed a significant higher TAC in the blue-grained Skorpion *cv* and a higher AC_FRAP_ in the yellow-grained Bona Vita *cv*. Conversely, no difference was observed for SPAs in any of the samples and for xanthophyll in Bona Vita *cv*. 

### 3.3. Gluten Aggregation Properties

**Figure 2:**
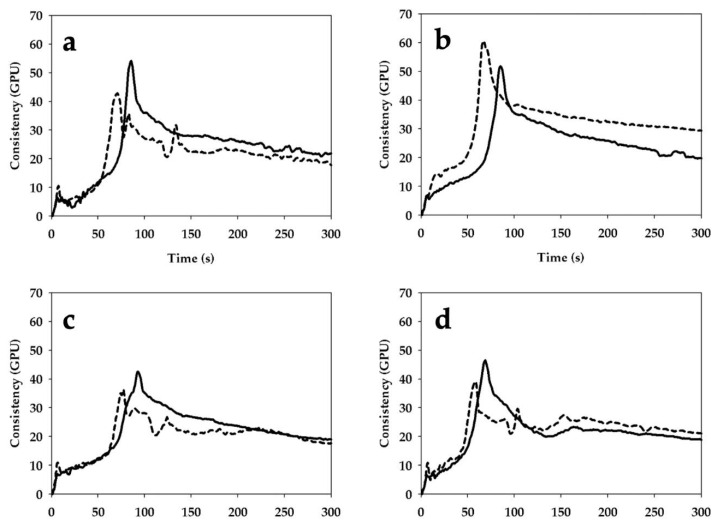
Effect of stone milling (solid line) and roller milling (dash line) on gluten aggregation properties, assessed by GlutoPeak^®^, of whole grain flours from Bolero *cv* (**a**), CWRS (**b**), Bona Vita *cv* (**c**) and Skorpion *cv* (**d**). CWRS: commercial Canada Western Red Spring Wheat; GPU: GlutoPeak Units.

The gluten aggregation profiles of the WFs were consistent in showing a slower aggregation when the samples were milled by stone milling (Figure 2). Specifically, the peak maximum time was significantly higher in SWFs than RWFs (86 vs. 71 s, 86 vs. 64 s, 101 vs. 79 s, and 72 vs. 58 s for Bolero, CWRS, Bona Vita, and Skorpion *cvs*, respectively). 

In the case of maximum torque and aggregation energy, the effect of the milling system depended on the type of wheat. Specifically, SWFs showed a significantly higher maximum torque than RWFs (55.1 vs. 43.7 GPU and 46.2 vs. 37.3 GPU for Bolero and Bona Vita *cvs*, respectively), as well as significantly higher aggregation energy (1220 vs. 1001 GPE, 1099 vs. 864 GPE, and 1080 vs. 908 GPE, for Bolero, Bona Vita and Skorpion *cvs*, respectively) (Figure 2a,c,d). On the contrary, significantly lower values for both indices were found in the case of CWRS (52.2 vs. 59.7 GPU and 1216 vs. 1387 GPE for maximum torque and aggregation energy, respectively) (Figure 2b).

### 3.4. Mixing Properties

**Figure 3:**
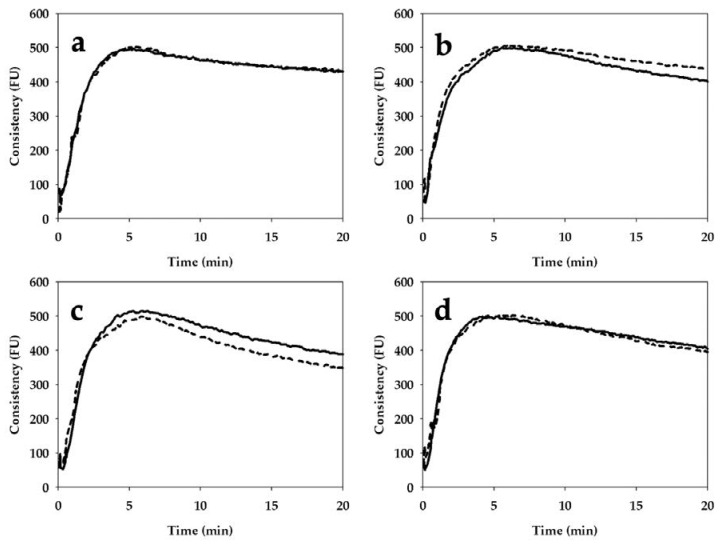
Effect of stone milling (solid line) and roller-milling (dash line) on the mixing properties, assessed by Farinograph^®^, of whole grain flours from Bolero *cv* (**a**), CWRS (**b**), Bona Vita *cv* (**c**) and Skorpion *cv* (**d**). CWRS: commercial Canada Western Red Spring Wheat; FU: Farinographic Units.

As regards the mixing properties (Figure 3), stone milling resulted in a significantly higher water absorption value for Bolero *cv* (61.7 vs. 64.2%, RWF vs. SWF) and Bona Vita *cv* (64.7 vs. 68.1% RWF vs. SWF). On the contrary, the milling system did not significantly affect the dough development time.

In addition, stone milling led to a significant increase in dough stability only in the case of Bolero *cv* (from 4.6 to 6.2 min for RWF and SWF). Finally, stone milling significantly influenced mixing resistance (evaluated as the degree of softening) only for CWRS and Bona Vita *cv*. Specifically, a higher degree of softening was observed in SWF (88 FU) compared to RWF (65 FU) in the case of CWRS, while this parameter decreased (from 138 to 119 FU, for RWF and SWF, respectively) in the case of Bona Vita *cv*.

### 3.5. Leavening Properties

**Figure 4:**
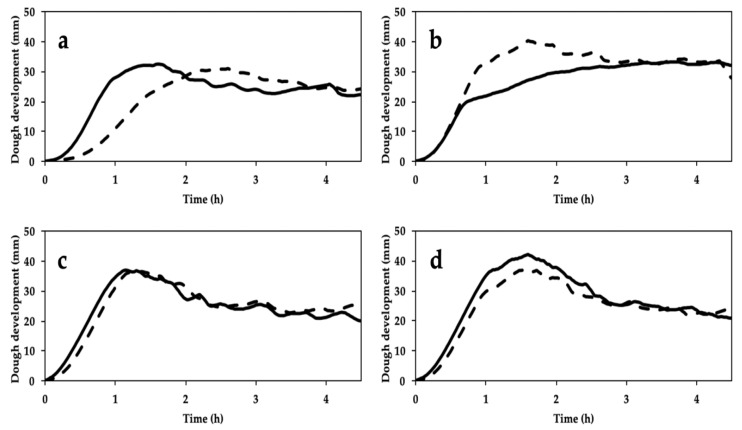
Effect of stone milling (solid line) and roller milling (dash line) on dough development, assessed by Rheofermentometer^®^, during leavening of whole grain flours from Bolero cv (**a**), CWRS (**b**), Bona Vita cv (**c**) and Skorpion cv (**d**). CWRS: commercial Canada Western Red Spring Wheat.

The greatest impact of stone milling on dough leavening properties was observed for CWRS and Bolero cv, but each sample exhibited a different trend (Figure 4). Specifically, in the case of CWRS, stone milling caused a decrease in maximum dough development (from 40 to 33 mm), instead an opposite trend was found for Skorpion cv (from 37 to 42 mm). Moreover, stone milling resulted in increased dough height at the end of the test for CWRS (from 24 to 33 mm), as opposed to decreased height for the Bona Vita cv (from 34 to 20 mm). Moreover, the time required to reach maximum dough development was lower in stone milling for Bolero cv (2.36 and 1.38 h, for SWF and RWF respectively), as opposed to an increase in this index for CWRS (1.36 and 4.49 h, for SWF and RWF respectively), whereas no differences were observed for Bona Vita and Skorpion cvs (Figure 4).

**Figure 5:**
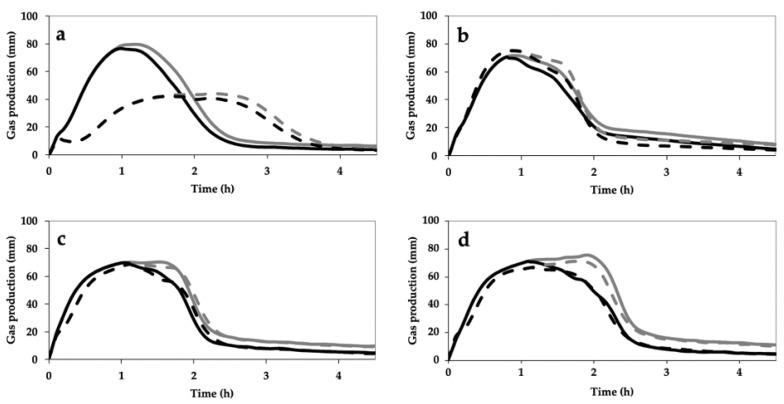
Effect of stone milling (solid line) and roller milling (dash line) on the total gas production (grey line) and on the retained gas (black line) in the dough, assessed by Rheofermentometer^®^, of whole grain flours from Bolero *cv* (**a**), CWRS (**b**), Bona Vita *cv* (**c**) and Skorpion *cv* (**d**).

As regards the volume of CO_2_ developed (Figure 5), stone milling resulted in an increase in this index for Bolero (from 1150 to 1398 mL) and Skorpion (from 1532 to 1659 mL) cvs. In addition, regardless of the type of wheat, stone milling led to an increase in the amount of CO_2_ released (from 124 to 197 mL, from 195 to 211 mL, from 191 to 205 mL, and from 260 to 332 mL for Bolero, CWRS, Bona Vita and Skorpion cvs, respectively). Finally, no difference was observed in terms of the retention coefficient between the two milling approaches for the four samples.

### 3.6. Bread Properties

**Figure 6:**
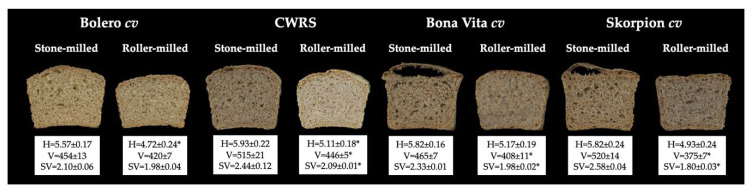
Effect of stone and roller milling on bread height (H; cm), volume (V; mL) specific volume (SV; mL/g). Data are presented as mean ± standard deviation. The asterisks indicate significant differences between the mean of the bread from stone and roller milled flours of each *cvs* (* *p* < 0.01). The absence of asterisk indicates a not significant difference. CWRS: commercial Canada Western Red Spring Wheat.

As for bread properties, stone milling resulted in higher bread height for Bolero *cv* and CWRS, instead no significant differences were observed for Bona Vita and Skorpion *cvs* (Figure 6). As regards volume and specific volume, bread from CWRS, Bona Vita and Skorpion *cvs* showed significant higher values when the stone milling system was used. However, bread from Bona Vita and Skorpion *cvs* exhibited a large bubble in the crumb layer under the upper surface of the bread, due to a collapse of the crumb structure. 

The analyses of bioactive compounds and AC were carried out on bread samples obtained from SWF, RWF and refined flours of pigmented *cvs*, chosen to provide more interesting phytochemical comparisons (Table 4). Compared to WFs, the removal of wheat bran led to a significant decrease in several antioxidant compounds both in refined flours and derived breads. On average, bread obtained from refined flours showed a lower content of CWBPA (−94%), SPA (−89%), AC_ABTS_ (−27%), AC_FRAP_ (−90%), zeaxanthin (−64%, Bona Vita *cv*) and TAC (−122 times, Skorpion *cv*). Conversely, lutein was higher (+29%) in bread from refined flour compared to WF samples. As highlighted in Table 4, bread samples obtained from the blue-grained *cv* showed levels of CWBPA (crumb and crust), SPA (crust), AC_FRAP_ (crumb) and TAC (crumb and crust) significantly higher for RWF as compared to SWF. RWF bread (crumb and crust) obtained from Bona Vita *cv* showed a significantly higher AC_FRAP_, but a significantly lower content of lutein, than SWF. No difference in the relative abundance of CWBPAs was observed by comparing refined flour, SWF and RWF: ferulic acid was in both flour and bread the most representative (88%), followed by sinapic acid (5%) and *p*-cumaric acid (4%). Conversely, as far as the SPAs were concerned, a different behavior was observed in all compared flours: bread-making increased the relative abundance of soluble ferulic acid (on average from 27% to 46%), while reducing the percentage of soluble sinapic acid (from 41% to 32%).

## 4. Discussion

Despite the numerous websites and skilled marketing operations that declare the uniqueness and authenticity of SWF, the effects of stone milling—in comparison with those promoted by roller milling—on the chemical, rheological, and bread-making properties of the related flours have not yet been investigated systematically. The present study seeks to fill this gap. Moreover, since differences in kernel characteristics affect the milling process, four types of wheat were chosen for their variations in hardness, gluten strength, and richness in biocomponents. Specifically, the two wheat samples frequently used in the bread sector were Bolero (soft white winter wheat *cv*) and a commercial Canada Western Red Spring (CWRS). The other two wheat *cvs* were Bona Vita (medium-hard winter wheat with yellow endosperm for the high xanthophyll content) and Skorpion (medium-hard winter wheat with a blue external layer, rich in anthocyanins).

There is a widespread belief among consumers that, from a nutritional point of view, SWF are better than RWF, thus the label “made with stone-ground flour” is a powerful marketing tool for both producers and retailers [32]. In accordance with the AACC International definition of whole grain [33], neither milling process selected the anatomical regions, and endosperm, bran and germ have to be present in the same proportions as in the intact caryopsis. In agreement with other authors [17,20,22], no significant changes in the proximate composition were found regardless of the milling process used (Table 3). The few differences between SWFs and RWFs concerned moisture, which was significantly higher in RWFs from Bona Vita and Skorpion *cvs*. This result might firstly be related to the lack of conditioning before stone milling; moreover, a drop of moisture might be associated with heat development during this milling process, as mentioned by several authors [14,15,17].

The total dietary fiber content of WFs was included in the range 9.3%–14.3% (Table 3), similar to that observed for WFs examined within the European HEALTHGRAIN Project [10]. Regarding the potential effects of milling process on fiber fraction content, the SWF and RWF of each wheat sample exhibited no differences in total, insoluble and soluble fractions related to the milling process. Regarding the arabinoxylan fraction, the differences in the amounts of total and water-extractable arabinoxylans did not show a common trend regardless of the milling process used (Table 3). Nevertheless, in the two pigmented *cvs*, both of these parameters were considerably higher than in Bolero *cv* and CWRS, highlighting that Skorpion and Bona Vita *cvs* contain other interesting nutritional traits in addition to polyphenols or xanthophylls suitable for their exploitation. Although the occurrence of several macronutrients was unchanged in the compared WFs, RWFs of pigmented *cvs* resulted in a higher content of antioxidant compounds than SWF. Carcea et al. [20] reported no compositional difference regarding the total polyphenol and alkylresorcinol contents between stone milled or roller milled flours. In our study, in particular, a significantly higher content of CWBPAs and TAC were present in RWFs. Results could be related to the higher heat generated during stone milling due to friction [15]. Prabhasankar and Rao [17] observed that the higher temperature detected in SWF (85 °C), resulted in protein degradation, a reduction of amino acid content and a loss of some essential fatty acid compared to RWF (32 °C). Similarly, by comparing a stone milling process (60 °C) to a watermill process able to generate lower temperatures (30 °C), Di Silvestro et al. [34] observed a decrease in bound phenolic fraction, while no effect was detected for arabinoxylans and β-glucans.

In conventional roller milling, the importance of kernel tempering (or conditioning) to guarantee high yield and high quality of flour is widely recognized [11,12,13]. Indeed, particle size distribution after the first break and, consequently, the behavior of the “broken material” in all the remaining milling passages, is strictly influenced by kernel moisture [35]. On the contrary, no mention is made about the need to modify the native moisture of kernels before stone milling [11,16]. Such differences, and specifically, the increased moisture of the pericarp assured by tempering before roller milling could be the main reason for the differences in particle size distribution between SWFs and RWFs (Figure 1). Indeed, by lowering the native friability of the bran layers, moistening facilitates their separation from the starchy endosperm in large flakes during roller milling. At the same time, the increased endosperm moisture—in respect to the native kernel—induces the efficacious breakage of this region, yielding a high percentage of fine particles (Figure 1). This pattern is congruent with the results of Kihlberg et al. [22] and Ross and Kongraksawech [14], the latter investigating eight different small-scale mills. Although a common trend in all RWFs is recognizable, the moisture distribution inside the kernels could not be optimal irrespective of their hardness, as tempering conditions were the same for all wheat samples (moisture before milling equal to 16%). Bearing in mind the results obtained by Doblado-Maldonado et al. [36], CWRS could present, particularly in the external layers, lower moisture than required for optimal roller milling; this physical condition might account for the low percentage of large bran particles (Figure 1). Roller milling of medium-hard (i.e., Bona Vita and Skorpion *cvs*) and soft (Bolero *cv*) wheat, likely optimally moistened, yielded WFs with similar particle size distribution, characterized by a high percentage of both coarse and fine particles. On the contrary, when stone milling was applied, due to its different breakage system and lack of conditioning before milling, bran and endosperm regions exhibited a similar behavior during the breakage actions. Consequently, particles in SWFs were more homogeneously distributed in classes of different size, particularly in medium-sized classes (from about 300 to 700 μm) (Figure 1): the pattern was similar for all varieties, regardless of their hardness.

Evaluation of particle size distribution is important for understanding the rheological properties of dough: indeed, the particle size of bran and/or flour influences several features, including water absorption and gluten aggregation kinetics. Nevertheless, the literature has yet to indicate the ‘optimal’ particle size distribution for bread-making [37,38].

Although the milling system did not affect the protein content of the WFs (Table 3), some changes in protein properties—that are important for bread-making performance—were highlighted by the rheological tests. The analysis of gluten aggregation properties by means of a rapid shear-based method (i.e., GlutoPeak test) indicated that gluten proteins were able to aggregate and show a peak (Figure 2), which represents the maximum extent of gluten formation before its breaking due to the intense shear-stress [39]. Overall, RWFs exhibited faster gluten aggregation (lower peak maximum time), required less energy to aggregate and resulted in lower maximum consistency (except for CWRS) than SWFs, suggesting gluten weakening. A similar trend has been observed while comparing refined and whole flours due to the interference of fiber in network formation [40,41]. In the case of RWFs, the weakening of the gluten network could be due to depolymerization phenomena, favored by the presence of free-SH groups, particularly abundant in WFs with coarse particles (average particle size: 830 μm) [42]. Certainly, the presence of high amounts (more than 15% *w/w*) of large particles (>1000 μm size) in RWFs from Bolero, Bona Vita and Skorpion *cvs* might have negatively affected protein-protein interactions via physical mechanisms [42]. Similarly, the low percentage (only 7%) of the same size class in the RWF from CWRS might account for its opposite performance: both maximum torque and aggregation energy exhibited higher values than those determined in SWF.

Moving to the mixing properties evaluated by the farinographic test (Figure 3), the milling process did not seem to have a conclusive effect on such properties, that are greatly affected by the type of wheat. As emphasized by Ross and Kongraksawech [14], the farinographic indices were primarily influenced by *cv* and less by the milling process. In contrast to the gluten aggregation kinetics (evaluated by the GlutoPeak test on a slurry), the mixing properties were evaluated on a dough applying lower stress to the system (63 rpm vs. 2750 rpm). Thus, the apparent different findings could be attributed to the differences in the test conditions.

In general, the water absorption index was higher in SWFs (in Bolero and Bona Vita *cvs*), probably as the consequence of their higher (although not significant) amounts of damaged starch (Table 3), as the role of bran particle size, proposed as a valid explanation by Kihlberg et al. [22], was not highlighted. Stability was significantly affected only for Bolero *cv* (6.2 and 4.6 min for SWF and RWF, respectively). An important role might be played by the distribution of large/coarse bran particles (>1000 μm size) (Figure 1) which were three times higher in the RWF of Bolero *cv*. They were probably responsible for the weakness in its gluten network and, consequently, the significant decrease in dough stability. Moreover, the high percentage of large bran particles could impair not only dough properties during bread making but also the bioavailability of minerals, as indicated by Miller Jones et al. [10]. Nevertheless, the role of bran particle size on dough and bread characteristics needs to be further investigated as the results of works on this subject are still contradictory [38].

Regardless of the milling process, Skorpion and Bona Vita *cvs* showed similar leavening profiles (Figure 4), in agreement with their similar proximate composition (Table 3) and trends observed through the GlutoPeak test (Figure 2) and the farinographic test (Figure 3). Anyway, Skorpion WFs (both SWF and RWF) resulted in good dough development (Figure 4d) and gas production (Figure 5g,h), likely due to the high dough stability as shown by the farinographic test.

Differences in rheological properties associated with the milling systems were evident only for Bolero *cv* and CWRS. As considering the indications of the other rheological tests, Bolero *cv* and CWRS showed an opposite behavior according to the milling process; moreover, despite a similar protein content (Table 3), these samples were characterized by relevant differences in protein quality (Figure 2). Specifically, as for Bolero *cv*, SWF reached similar dough heights than RWF but faster, probably due to its higher—although not significant—damaged starch content as a quick source of simple sugars for yeast growth. In addition, RWF produced the least gas (Figure 5b) and the lowest bread volume (Figure 6). Both leavening properties and bread-making performance might be due to the high percentage of large particle size in RWF in Bolero *cv* (Figure 1). As expected, RWF of CWRS performed best during leavening in terms of dough development and time to reach it, indicative of the ability of this wheat type to withstand leavening stresses. Other reasons which might account for this result include good gluten aggregation (Figure 2) and mixing (Figure 3) properties, associated with the high damaged starch content and the low fiber percentage (Table 3) among the samples considered in this study.

Among the rheological tests used to predict the bread-making performance of samples, only the GlutoPeak tests (Figure 2) agreed for all samples as volume, specific volume and height of bread (Figure 6). Indeed, for all the wheat types, both the loaf height and the specific volume of bread samples produced with SWFs were higher than those obtained from RWFs, in agreement with the observations by Kihlberg et al. [22]. Our findings were also congruous with those of Gélinas et al. [18] which showed that dough mixing properties of WFs—in terms of farinographic absorption and stability—did not always relate to bread properties and, therefore, did not explain why some varieties performed better than others.

As the characteristics of bread are related not only to dough properties—generally evaluated by tests carried out at temperatures below 30 °C—but also to phenomena occurring during baking, we can hypothesize that, during baking, proteins in dough from SWFs might have retained extensibility for a longer time, assuring a higher bread volume. According to the literature [14,22,38,43,44], particle size distribution of WF might represent another trait able to influence bread volume. As previously discussed, stone milling produced a large amount of medium-coarse particles (from 300 to 700 μm) (Figure 1) that, according to Doblado-Maldonado et al. [15], could be considered the most advantageous for bread production. The particle size distribution observed in RWF (especially fine and large particles, simultaneously) (Figure 1) accounted for the low bread development. Indeed, small particles (<250 μm) could have a negative effect on bread characteristics as they promptly interfere in protein-protein interactions due to their high contact surface [43]. Also large particles might exert an undesirable action towards gluten development and gas cell stabilization [42] and bread appearance and texture [45].

Despite their high volume, in the case of Bona Vita and Skorpion *cvs*, stone milling resulted in a huge bubble just under the crust, together with the collapse of the underlying crumb (Figure 6). These behaviors cannot be attributed to differences in α-amylase activity (data not shown). The pattern of the corresponding rheofermentograph traces (Figure 4) allows us to hypothesize that, at the beginning of baking, the gas produced by yeasts in large amounts was not efficaciously held inside the gluten network and gathered in the upper part of loaf, causing the formation of a big bubble and a partial collapse of the underlying region.

The slight but significantly higher content of bioactive compounds in RWF flour compared to SWF (Table 3) was confirmed after bread-making. In particular, a higher CWBPA and TAC content found in both RWF bread crumb and crust for the blue-grained *cv*, resulting in a higher AC (FRAP assay, Table 4). As observed in previous studies [46,47], during bread-making significant changes occur in both bioactive compounds and AC. In the present study, bread-making caused a significant loss of the antioxidants responsible for the grain and flour pigmentation (xanthophylls and anthocyanins). Nevertheless, an increase in the AC was observed in the bread crust (Table 4). This could be due to the neo-formation of Maillard reaction products [46,48].

## 5. Conclusions

Most consumers believe that only the stone-milling process is able to preserve all the nutrients and bioactive compounds of wheat grains as, in this process, all kernel regions form a single stream. Indeed, the roller-milling process (a multiple-stream approach where the fractions must be recombined to obtain WF) is wrongly but commonly associated with a partial depletion of the native nutrients of the kernel. Our results proved that SWFs have neither a better proximate composition, nor a better bioactive compound concentration than RWFs. Only for blue-grained *cv* (Skorpion) RWFs resulted in a slight but significant higher content of CWBPAs and TAC compared to SWFs and this feature was observed also in bread.

The comparison of SWF and RWF properties highlighted a different particle size distribution. Indeed, during the grinding of caryopsis through the stone or the roller-milling, compression, shear, and cutting stresses exhibited different intensities and degrees (due to intrinsic kernel factors and process conditions), promoting the formation of large bran particles and very small flour particles in RWFs, while a more homogeneous particle size distribution was observed in SWFs. Although it can be assumed that these physical features could greatly affect the surface properties and the hydration properties of flour, only the GlutoPeak test, a quite recent rheological approach proposed for evaluating the protein-protein aggregation kinetics in wheat, highlighted significant differences in the gluten properties of WFs according to the milling process which were congruent with their bread-making performances.

The rheological differences between the WFs obtained from stone- or roller-milling, although significant, do not make one process clearly preferable to the other one. However, further information on the sensory profile of bread is worthy of interest. Nevertheless, the lower productivity of the former is acceptable for artisan or home-made processes, while the higher flexibility and versatility of multiple-stream roller-milling, and its fully automated management, can better satisfy industrial purposes. On the other hand, the effect of heat treatments (for stabilizing bran and germ) on the nutritional features of RWFs should be considered, as well as the effect of re-milling large bran particles on the technological performances.

## Figures and Tables

**Table 1:** Main information of common wheat samples.

*Cultivar*	Cultivation Region	Hardness	Color	TW kg/hL	TKW g
Bolero	Piedmont region, Italy	soft	white	78.0	33.3
CWRS	Manitoba region, Canada	hard	red	77.8	34.8
Bona Vita	Piedmont region, Italy	medium	yellow	74.6	36.5
Skorpion	Piedmont region, Italy	medium	blue	70.4	48.0

TW, test weight; TKW, thousand kernel weight.

**Table 2:** Amount of water, kneading and leavening times used in bread-making for each sample.

	Bolero *cv*		CWRS		BonaVita *cv*		Skorpion *cv*	
	Stone-Milling	Roller-Milling	Stone-Milling	Roller-Milling	Stone-Milling	Roller-Milling	Stone-Milling	Roller-Milling
Amount of tap water (g/100 g of flour)	64.2	61.7	61.2	61.5	68.1	64.7	65.5	64.5
Kneading time (min)	5.0	5.5	6.7	6.0	5.6	5.9	4.3	4.9
Leavening time (h)	1.38	2.36	1.44	1.36	1.09	1.15	1.36	1.31

**Table 3:** Chemical composition and bioactive compounds of whole flours obtained by stone-milling and roller-milling.

	Bolero *cv*		CWRS		Bona Vita *cv*		Skorpion *cv*		Average of All Samples	
	Stone-Milling	Roller-Milling	Stone-Milling	Roller-Milling	Stone-Milling	Roller-Milling	Stone-Milling	Roller-Milling	Stone-Milling	Roller-Milling
Moisture	11.8 ± 0.1	13.2 ± 0.7	14.8 ± 0.1	14.9 ± 0.7	11.8 ± 0.1	14.2 ± 0.1 *	12.4 ± 0.2	13.9 ± 0.1 *	12.7	14.05
Protein	16.7 ± 0.3	16.8 ± 0.2	16.9 ± 0.2	16.8 ± 0.2	15.4 ± 0.2	15.9 ± 0.2	15.3 ± 0.3	15.8 ± 0.8	16.1	16.3
Ash	1.5 ± 0.1	1.8 ± 0.1	1.5 ± 0.1	1.3 ± 0.1	1.7 ± 0.2	2.0 ± 0.2	1.7 ± 0.2	1.6 ± 0.1	1.6	1.7
Fat	1.8 ± 0.1	2.1 ± 0.01	1.9 ± 0.002	2.3 ± 0.1	2.4 ± 0.1	2.2 ± 0.04	1.9 ± 0.2	2.3 ± 0.3	2.0	2.2
Starch										
Total	66.5 ± 0.7	65.9 ± 0.3	67.9 ± 1.0	70.4 ± 0.4	66.8 ± 0.2	65.6 ± 0.8	68.5 ± 0.2	67.5 ± 0.5	67.4	67.4
Damaged	3.3 ± 0.1	2.2 ± 0.3	5.0 ± 0.5	6.2 ± 0.5	4.0 ± 0.3	3.5 ± 0.3	4.7 ± 1.0	4.3 ± 0.4	4.3	4.1
Dietary Fiber										
Total	13.4 ± 0.4	13.4 ± 0.1	11.7 ± 1.2	9.3 ± 0.2	13.6 ± 0.1	14.3 ± 0.4	12.5 ± 0.2	12.8 ± 0.2	12.8	12.5
Insoluble	11.2 ± 0.01	11.5 ± 0.2	9.8 ± 0.6	7.0 ± 0.5	11.8 ± 0.1	12.1 ± 0.2	10.6 ± 0.3	10.6 ± 0.3	10.9	10.3
Soluble	2.2 ± 0.4	1.9 ± 0.3	1.9 ± 0.6	2.3 ± 0.3	1.8 ± 0.2	2.2 ± 0.2	1.9 ± 0.1	2.2 ± 0.2	2.0	2.2
Arabinoxylan										
Total	4.0 ± 0.3	3.7 ± 0.12	3.74 ± 0.04	3.04 ± 0.05 *	5.44 ± 0.20	4.79 ± 0.01 *	4.01 ± 0.40	6.8 ± 0.2 *	4.3	4.6
Water extractable	0.23 ± 0.01	0.19 ± 0.05	0.33 ± 0.02	0.29 ± 0.02	0.32 ± 0.02	0.52 ± 0.01 *	0.50 ± 0.05	0.44 ± 0.01	0.35	0.36
CWBPAs	606 ± 42	626 ± 30	639 ± 54	684 ± 10	766 ± 16	886 ± 1 7 *	897 ± 19	991 ± 7 *	727	797
CWB-Ferulic acid	551 ± 39	570 ± 28	591 ± 52	629 ± 9	672 ± 16	783 ± 12 *	792 ± 17	874 ± 6 *	652	714
CWB-Sinapic acid	24.8 ± 2.3	25.5 ± 1.8	22.4 ± 0.9	26.4 ± 0.9 *	50.5 ± 2.1	53.1 ± 6.5	46.2 ± 0.4	48.8 ± 1.9	36.0	38.5
SPAs	52.8 ± 3.9	52.2 ± 3.4	59.0 ± 5.5	56.9 ± 1.5	62.1 ± 5.2	74.5 ± 1.0	84.7 ± 13.7	91.7 ± 2.6	64.7	68.8
S-Ferulic acid	13.9 ± 0.9	13.9 ± 1.0	15.8 ± 1.9	14.9 ± 0.6	15.7 ± 2.2	18.7 ± 1.4	20.8 ± 3.4	23.1 ± 1.0	16.6	17.7
S-Sinapic acid	24.1 ± 1.4	23.7 ± 1.6	29.5 ± 2.7	29.1 ± 0.7	32.2 ± 2.0	38.4 ± 2.6	43.4 ± 7.1	45.2 ± 1.4	32.3	34.1
Xanthophylls										
Lutein	n.d.	n.d.	n.d.	n.d.	3.3 ± 0.1	3.0 ± 0.2	n.d.	n.d.	-	-
Zeaxanthin	n.d.	n.d.	n.d.	n.d.	0.28 ± 0.02	0.24 ± 0.02	n.d.	n.d.	-	-
TAC	n.d.	n.d.	n.d.	n.d.	n.d.	n.d.	22.7 ± 0.4	26.3 ± 0.2 *	-	-
AC_ABTS_	20.3 ± 0.3	20.2 ± 0.3	19.2 ± 0.3	19.1 ± 1.1	18.2 ± 0.8	18.0 ± 0.4	19.9 ± 0.7	18.6 ± 0.3	19.4	19.0
AC_FRAP_	7.3 ± 0.5	6.7 ± 0.4	6.3 ± 0.4	6.4 ± 0.2	6.4 ± 0.1	8.1 ± 0.4 *	7.9 ± 0.6	7.3 ± 0.2	7.0	7.1

Protein, ash, fat, total dietary fiber and total arabinoxylans values are expressed as g/100 g dry basis. Damaged starch is expressed as g/100 g of total starch. Insoluble and soluble dietary fiber are reported as g/100 g dry basis of total dietary fiber. Water-extractable arabinoxylans are expressed as g/100 g dry basis the total arabinoxylans. Cell wall-bound phenolic acids (CWBPAs) and soluble (free and conjugated forms) phenolic acids (SPAs) are the sum of the single phenolic acids determined by means of RP-HPLC/DAD and are expressed as mg/kg dry basis. Xanthophyll (lutein and zeaxanthin) are expressed as mg/kg dry basis. Total anthocyanin content (TAC) is expressed as mg Cy-3-glc eq/kg dry basis. Antioxidant capacity (AC) measured by means of the ABTS and FRAP assays is expressed as mmol TE/kg dry basis. CWRS: commercial Canada Western Red Spring Wheat. Data are presented as mean ± standard deviation. The asterisks indicate significant differences between the means of stone- and roller-milled samples of each *cv* (* *p* < 0.01). The absence of asterisk indicates a not significant difference. n.d.: not determined.

**Table 4:** Cell wall-bound phenolic acids (CWBPAs), soluble phenolic acids (SPAs), antioxidant capacity (AC), xanthophylls (lutein and zeaxanthin) and total anthocyanin content (TAC) in raw material (flour), bread crust and bread crumb obtained from refined white flour, stone (SWF) and roller-milled (RWF) whole-grain flour of Bona Vita and Skorpion *cvs*.

*cv*	Flour	Product	CWBPAs	SPAs	Lutein	Zeaxantin	TAC	AC_ABTS_	AC_FRAP_
Bona Vita	refined	raw material	56.4 c	9.6 d	4.0 a	0.16 c	n.d.	13.5 b	0.2 g
		bread crumb	52.6 c	10.1 d	1.9 d	0.08 e	n.d.	12.8 bc	0.2 g
		bread crust	59.4 c	9.6 d	1.4 e	0.07 e	n.d.	11.3 c	1.8 f
	SWF	raw material	765.5 b	62.1 c	3.3 b	0.28 a	n.d.	18.2 a	6.4 e
		bread crumb	774.4 b	79.7 ab	1.5 e	0.14 cd	n.d.	17.5 a	7.5 d
		bread crust	770.2 b	76.6 b	1.0 f	0.12 d	n.d.	17.5 a	13.2 b
	RWF	raw material	885.7 a	74.5 b	3.0 c	0.24 b	n.d.	18.0 a	8.1 cd
		bread crumb	795.9 b	86.0 a	1.2 f	0.11 d	n.d.	17.2 a	8.8 c
		bread crust	808.6 ab	73.8 b	1.0 f	0.12 d	n.d.	17.6 a	15.9 a
Skorpion	refined	raw material	50.3 d	8.4 e	n.d.	n.d.	0.1 e	14.8 d	0.2 e
		bread crumb	56.8 d	12.3 e	n.d.	n.d.	0.1 e	13.4 e	0.5 e
		bread crust	59.2 d	10.3 e	n.d.	n.d.	0.1 e	10.8 f	3.3 d
	SWF	raw material	897.4 b	84.7 cd	n.d.	n.d.	22.8 b	19.9 a	7.9 c
		bread crumb	781.4 c	95.5 abc	n.d.	n.d.	11.2 d	17.2 c	8.2 c
		bread crust	822.5 c	75.7 d	n.d.	n.d.	10.7 d	17.2 c	13.7 a
	RWF	raw material	990.5 a	91.7 bc	n.d.	n.d.	26.2 a	18.6 ab	7.3 c
		bread crumb	929.6 ab	107.2 a	n.d.	n.d.	13.3 c	18.1 bc	9.9 b
		bread crust	938.7 ab	98.9 ab	n.d.	n.d.	13.9 c	17.9 bc	13.6 a

Cell wall-bound phenolic acids (CWBPAs) and soluble (free and conjugated forms) phenolic acids (SPAs) are the sum of the single phenolic acids determined by means of RP-HPLC/DAD and are expressed as mg/kg dry basis. Xanthophylls (lutein and zeaxanthin) are expressed as mg/kg dry basis. Total anthocyanin content (TAC) is expressed as mg Cy-3-glc eq/kg dry basis. Antioxidant capacity (AC) measured by means of the ABTS and FRAP assays is expressed as mmol TE/kg dry basis. For each *cv*, value followed with different letters are significantly different (one-way ANOVA, *p* < 0.01), according to the REGW-F test.
